# Quasistatic Analysis of Precast Segmental Concrete-Filled Steel-Tube Bridge Pier with External Arched Energy Dissipation Device

**DOI:** 10.3390/ma16010340

**Published:** 2022-12-29

**Authors:** Chengquan Wang, Yanwei Zong, Yun Zou, Yonggang Shen, Jiqing Jiang, Chongli Yin

**Affiliations:** 1 Department of Civil Engineering, Zhejiang University City College, Hangzhou 310015, China; 2 Zhejiang Engineering Research Center of Intelligent Urban Infrastructure, Hangzhou 310015, China; 3 Key Laboratory of Safe Construction and Intelligent Maintenance for Urban Shield Tunnels of Zhejiang Province, Hangzhou 310015, China; 4 School of Environment and Civil Engineering, Jiangnan University, Wuxi 214122, China; 5 Department of Civil Engineering, Zhejiang University, Hangzhou 310058, China

**Keywords:** bridge seismic resistance, precast segmental concrete-filled steel-tube bridge pier, arched energy dissipation device, post-earthquake repair, quasistatic analysis

## Abstract

In order to further promote the application of segment-assembled bridge piers in medium- and high-intensity areas, and to reduce the post-earthquake damage and repair cost of bridge piers, in this paper, a precast segmental concrete-filled steel-tube bridge pier (PSCFSTBP) with an external arched energy dissipation device (AEDD) is proposed. Firstly, the effectiveness of the finite-element analysis software ABAQUS 6.14-4 is proved by the test results of the PSCFSTBP and the corresponding finite-element model analysis results. Secondly, ABAQUS 6.14-4 was used to establish four-segment PSCFSTBP models with four different structural forms (non-energy dissipation device, external arch steel plate, external vertical steel plate, and external AEDD), and the seismic performance of each model was compared and analyzed under reciprocating displacement loading. The results show that compared with the PSCFSTBP with an external AEDD, the lateral bearing capacity of the PSCFSTBP with an external vertical steel plate is increased by about 11.9%, and the initial stiffness is increased by about 2.5%. Compared with the PSCFSTBP with an external arch steel plate, the lateral bearing capacity, initial stiffness, and energy dissipation capacity are increased by 28.8%, 4.6%, and 13 times, respectively. Compared with the PSCFSTBP without an energy dissipation device, its lateral bearing capacity, initial stiffness, and energy dissipation capacity are increased by 39.4%, 10.4%, and 18 times, respectively. The residual displacement of the PSCFSTBP with an external AEDD is kept within 1 mm in the whole displacement loading stage, the offset rate is less than 1%, and the pier damage is controllable, which can realize rapid repair after an earthquake. Finally, the multi-level energy consumption and local replacement of the AEDD are also explored.

## 1. Introduction

In recent years, with the development of bridge technology, segmental prefabrication and assembly technology has not only been applied to the superstructure of the bridge, but also gradually applied to the pier structure [1]. Precast segmental piers can effectively shorten the construction period, improve construction efficiency and quality, and reduce traffic interference as well as construction cost [2,3].

However, precast segmental piers have limited application in areas with high seismic activity, and precast segmental piers are mostly used on highway bridges or canal bridges in low-intensity areas [4]. The rocking joints of prestressed segmental assembled piers can open and close during an earthquake, and reset through prestressed tendons with tensile force. These are called rocking self-resetting piers [5,6]. However, the whole confinement of precast segmental piers is insufficient [7]. The concrete at the joint may be under large compressive stress, which leads to premature crushing of the concrete, and the damage is mainly concentrated at the joint, especially the joint between the cushion cap and the bottom segment [2]. Although this can reduce the damage to other parts of the pier body, it also has a great impact on the self-resetting ability of the pier. Therefore, precast segmental assembled piers have limited application in areas with medium and high seismic activity.

Scholars at home and abroad have carried out a series of research to reduce the damage to precast segmental piers under earthquake conditions and improve the self-resetting ability of such piers. For example, Mander et al. [8] proposed the concept of “damage prevention design” by embedding 76 mm thick steel plates between piers and cushion caps, and setting rubber plates on the steel contact interface to form a rocking mechanism. It was found that the concrete damage at the joints of the segmental assembled piers and the residual displacement of the piers were significantly reduced, and the self-resetting ability of the piers was improved to a certain extent. Hews et al. [9] applied a wrapped steel tube to the bottom segment of a precast segment assembly pier. By quasistatic testing, it was found that the wrapped steel tube could form a constraint on the pier concrete to strengthen and avoid local damage to the concrete in the plastic hinge region, and the segmental assembly pier had little residual displacement and self-resetting ability. However, since the steel tube is only set in the plastic hinge region in the bottom part of the segment, the plastic length will increase and cause more serious damage to the upper segment. From previous work, it can be found that the application of a concrete-filled steel tube to segmental precast and assembled piers can avoid the local damage to the segment interface to a certain extent, and reduce the residual displacement of piers. In this regard, some scholars have studied segmental precast concrete-filled steel tubular piers and improved their seismic performance through external energy dissipation devices. Based on the research of Hews [9] et al., Chou et al. [10] set a precast segment as a concrete-filled steel tube, and set energy dissipation devices at the segment joints. According to the results of quasistatic tests, it was found that segmental assembled concrete-filled steel tube piers have excellent ductility and self-resetting capacity, with less degradation of residual displacement and stiffness, and external energy dissipation devices provide effective energy dissipation. Junfeng et al. [11,12] proposed bolted segmental precast concrete-filled steel tube piers, which are anchored by multiple bolts. The pseudo-static test results show that bolted precast segmental precast concrete-filled steel tube piers have good horizontal bearing capacity, small residual displacement, and external bolt connection can be repaired easily after a seismic event.

In recent years, as the seismic design concept of bridges has gradually changed from seismic mitigation to damage controllable, the life-cycle performance control of bridge structures considering the quick function recovery after a severe earthquake has become a new direction of bridge seismic design [13]. Han et al. [14] established 1/3-scale precast segmental assembled double-column piers with replaceable and proportionally reduced energy dissipation devices outside the bottom of piers. By pseudo-static testing, it was found that external energy dissipation devices improved the energy dissipation capacity of the pier, and the damage to the pier bottom was small. The plastic damage was concentrated on the energy dissipation devices, which could achieve rapid repair after an earthquake. Wang et al. [15,16,17] installed a replaceable energy dissipation device in the plastic hinge area of a hollow segmental pier, and assembled a replaceable energy dissipation reinforcement system using ultra-high-performance concrete slabs. Through tests, they found that the replaceable energy dissipation device is the main damage area, with controllable damage and easy post-earthquake repair. Moustafa et al. [18] controlled the damage to bridge piers by applying steel tubes to each segment of precast and assembled piers and adding energy dissipation devices at the joints. By shaking table tests, it was found that the damage to bridge piers was controllable, and the residual displacement was small. Chengquan et al. [19,20] found that there was no obvious damage to the pier shaft, plastic damage concentrated in the middle area of the energy dissipation device, the damage was controllable, and the residual deviation rate was within 1%, by setting steel energy dissipation devices at the joints of precast concrete-filled steel tubular piers and conducting low-cycle reciprocating loading tests.

To address the above problems, this study proposes an external arch energy dissipation device (AEDD) to improve the overall seismic performance of a precast segmental concrete-filled steel-tube bridge pier (PSCFSTBP) and achieve rapid repair after an earthquake. By establishing four different types of PSCFSTBP models, the seismic performance of the PSCFSTBP is compared and analyzed, the structural form of the AEDD is determined, the seismic mechanism of the PSCFSTBP with an external AEDD is explored, and the multi-level energy consumption mechanism and local interchangeability of the AEDD are analyzed.

## 2. Design of AEDD

**Figure 1:**
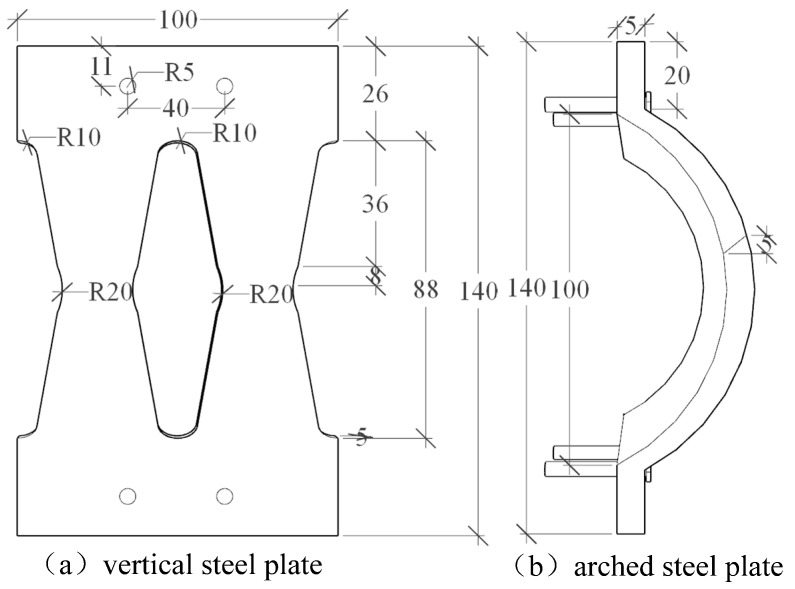
Model size of energy-consuming device (unit: mm).

**Figure 2:**
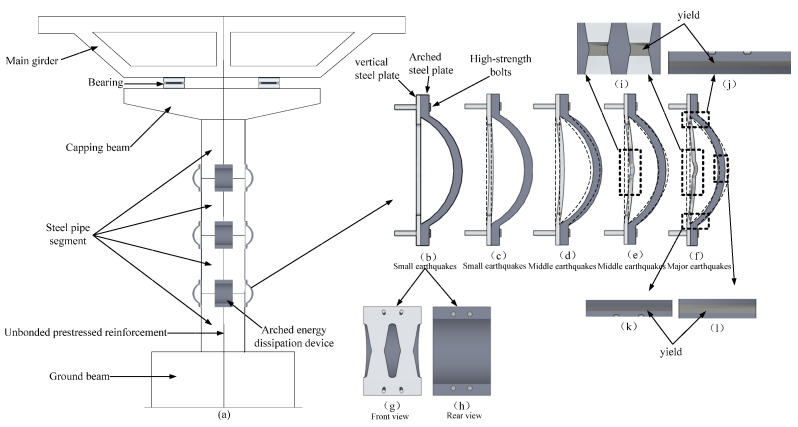
Schematic diagram of the force and failure process of the arched energy-consuming device.

The AEDD mainly includes a vertical steel plate and an arch steel plate. The device dimensions and component names are shown in Figure 1. In order to make full use of the energy consumption capacity of the AEDD, the vertical steel plate is made of low-yield-point (Q195) mild steel with less carbon content. In order to prevent damage at the AEDD bolt hole, a diamond-shaped hole is opened in the middle of the steel plate to make it a weak position, which can control the damage and facilitate replacement. The arch steel plate is a Q235 steel plate. The middle of the steel plate is precast into an arch by using a bending machine, and the vertical steel plate and the arch steel plate are connected to the PSCFSTBP with high-strength bolts (M24, Grade 10.9), as shown in Figure 2a. The AEDD is set at the seam of each section.

The vertical steel plate and the arched steel plate have the same projection size and bolt-hole position, which not only ensures that the two can be assembled and combined into AEDD, but also that the peeling of both can be placed on the same PSCFSTBP as an energy-dissipating element. Among them, the yield strength of the arched steel plate is greater than that of the vertical steel plate, but the weakening of the hole in the middle area of the vertical steel plate can concentrate the stress and control the damage.

Under horizontal seismic force, the AEDD at the tension side of this type of pier will produce the deformation process, as shown in Figure 2. When the horizontal offset of the pier top is 0–0.1%, the vertical steel plate and arch steel plate are free of deformation, as shown in Figure 2b. Figure 2g,h shows the front and rear views of the AEDD, respectively. With the increase in horizontal force, when the offset rate is 0.1–0.7%, the vertical steel plate begins to deform, but does not yield, while the arch steel plate has no obvious deformation, as shown in Figure 2c,d. When the offset rate is 0.7–1%, the vertical steel plate will yield, as shown in Figure 2e. Figure 2i shows the yield area of the vertical steel plate. It can be seen that the AEDD damage is concentrated in the middle weak position of the vertical steel plate, and the damage is controllable. The arch steel plate has a small deformation, and the AEDD starts to consume energy at the second level. When the offset rate is 1–1.3%, the arch steel plate reaches the yield strength, as shown in Figure 2f. Figure 2j–l shows the yield areas of the arch steel plate, respectively.

## 3. Finite-Element Numerical Simulation and Test Verification

### 3.1. PSCFSTBP Finite-Element Model Verification

In order to verify whether the various PSCFSTBP analysis models established by ABAQUS 6.14-4 are reliable, this section compares and analyzes the PSCFSTBP test results of the external diamond-shaped opening energy dissipation device [19] obtained from this test and the numerical analysis results of the corresponding finite-element model established, thus proving the accuracy of the parameter selection and simulation results of the finite-element model.

**Figure 3:**
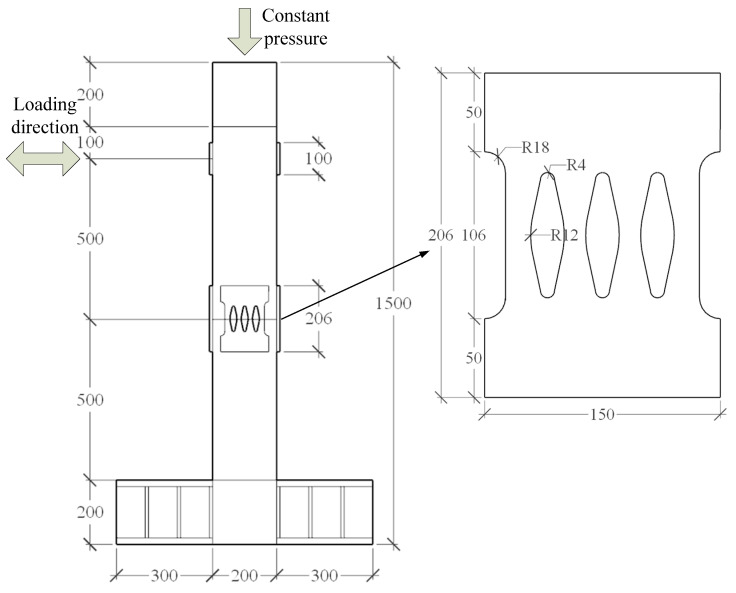
Dimensions of the specimen of the precast assembled bridge pier (unit: mm).

**Figure 4:**
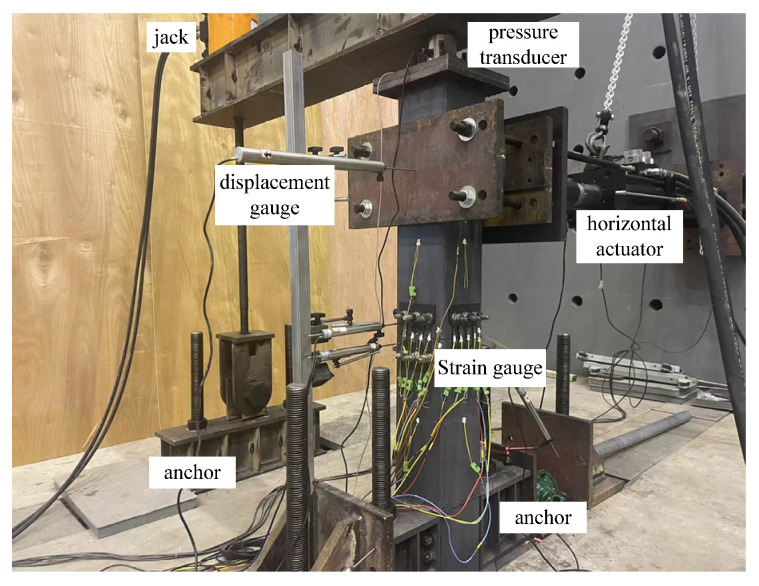
Experimental construction of precast assembled piers.

The piers in this test are composed of segmental steel tubes, core concrete, unbonded prestressed tendons, energy dissipation devices, etc., as shown in Figure 3 and Figure 4. Table 1 shows the performance of the materials. There are two equal-height precast steel tube confined concrete segments in the pier shaft. The height of each segment is 500 mm, and the rectangular section is adopted. The section size is 200 × 200 mm, 20 mm thick steel tube, Q345 steel, filled with C40 concrete. The energy dissipation element is made of Q235 steel with a thickness of 10 mm. The prestressed reinforcement is 4 steel strands with 7 strands of 15.2 mm, and the prestress applied is 200kN.

According to the deformation and stress characteristics of the model, the elastic–plastic model [21] is for the steel tube, steel strand, energy dissipation device, and reinforcement, and the constitutive relationship expression is as follows:

$$\sigma =\left\{ \begin{array}{l} E_{S}\varepsilon ,\\ f_{y}, \end{array}\right.\begin{array}{l} \varepsilon \leq \varepsilon_{y}\\ \varepsilon >\varepsilon_{y} \end{array}\}$$

where $E_{s}$ is the elastic modulus of steel.$\;f_{y},\;\varepsilon_{y}$ is the yield strength and corresponding yield strain of steel. *f*, *ε* is the steel stress and corresponding strain.

**Figure 5:**
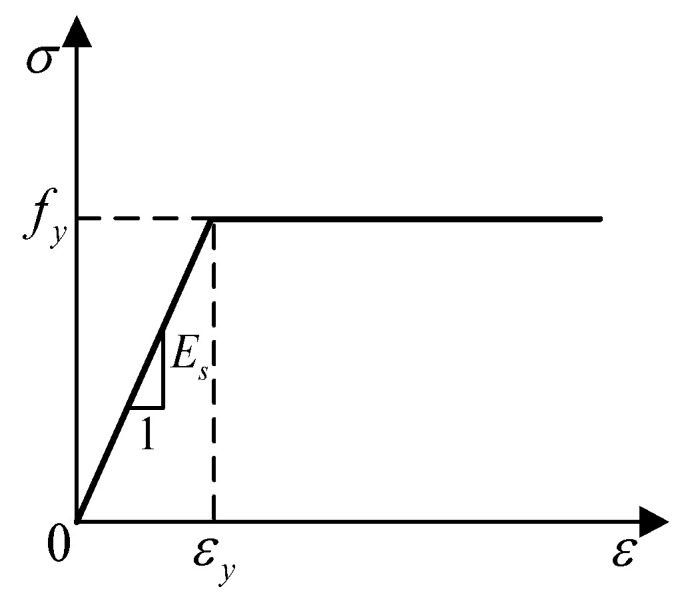
Stress–strain curves of steel.

The stress–strain relationship is shown in Figure 5.

For concrete, there are two types of constitutive relations between the outer concrete and core concrete of segmental precast concrete-filled steel tubular piers.

**Figure 6:**
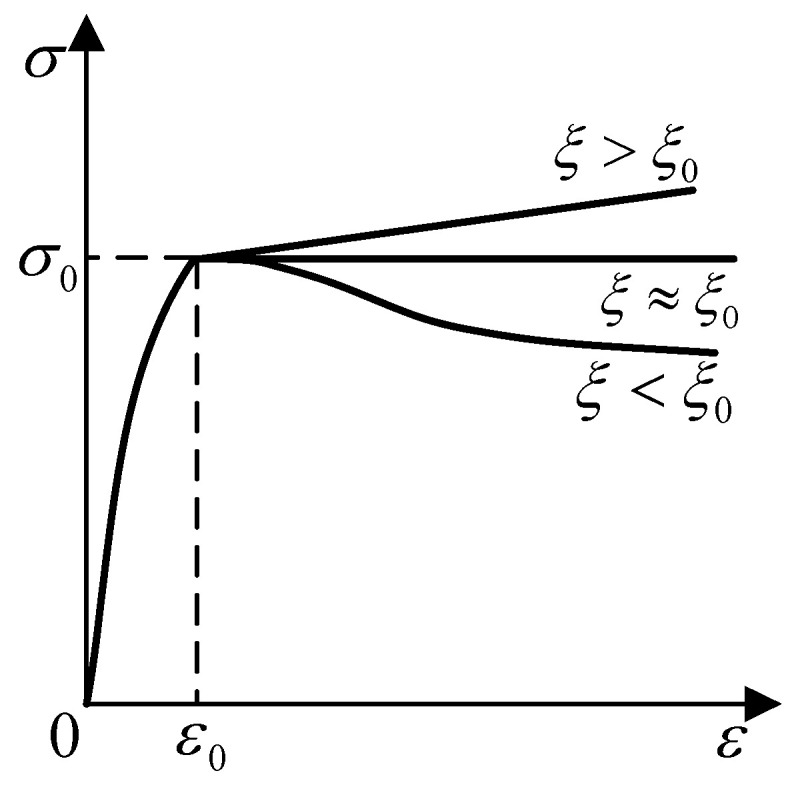
Stress–strain relationship of core concrete.

The stress–strain relationship proposed by Han Linhai [22] is for the core concrete, as shown in Figure 6, the expression is as follows:

$$\begin{array}{l} y=2x-x^{2}\;(x\leq 1)\\ y=\left\{ \begin{array}{ll} 1+q\cdot (x^{0.1\xi }-1) & \left(\xi \geq 1.12\right)\\ \frac{x}{\beta \cdot {\left(x-1\right)}^{2}+x} & \left(\xi <1.12\right) \end{array}\right.\;(x>1) \end{array}$$

where $x=\frac{\varepsilon }{\varepsilon_{0}}$, $y=\frac{\sigma }{\sigma_{0}}$, ε , and σ are the strain and stress of the concrete, and $\varepsilon_{0},\;\sigma_{0},\;q,\;\beta$ are calculation parameters.

The CDP damage plasticity model [23,24] is for the damage of external concrete in the process of stressing, and its tensile stress–strain expression is as follows:

$$\begin{array}{l} \sigma =(1-d_{t})E_{c}\varepsilon \\ d_{t}=\left\{ \begin{array}{ll} 1-\rho_{t}(1.2-0.2x^{5}) & \left(x\leq 1\right)\\ 1-\frac{\rho_{t}}{\alpha_{t}{\left(x-1\right)}^{1.7}+x} & \left(x>1\right) \end{array}\right.\\ X=\frac{\varepsilon }{\varepsilon_{t,r}}\\ \rho_{t}=\frac{f_{t,r}}{E_{c}\varepsilon_{t,r}} \end{array}$$

where $\alpha_{t}$ is the descent phase parameter, $f_{t,r}$ is the uniaxial tensile strength, $\varepsilon_{t,r}$ is the corresponding strain.

The relationship between compressive stress and strain is as follows:

$$\begin{array}{l} \sigma =(1-d_{c})E_{c}\varepsilon \\ d_{c}=\left\{ \begin{array}{ll} 1-\frac{\rho_{c}n}{n-1+x^{n}} & \left(x\leq 1\right)\\ 1-\frac{\rho_{c}}{\alpha_{c}{\left(x-1\right)}^{2}+x} & \left(x>1\right) \end{array}\right.\\ \rho_{c}=\frac{f_{c,r}}{E_{c}\varepsilon_{c,r}}\\ n=\frac{E_{c}\varepsilon_{c,r}}{E_{c}\varepsilon_{c,r}-f_{c,r}}\\ X=\frac{\varepsilon }{\varepsilon_{c,r}} \end{array}$$

where $\alpha_{c}$ is the parameter in the descending stage, $f_{c,r}$ is the uniaxial compressive strength, and $\varepsilon_{c,r}$ is the corresponding strain.

Table 2 shows specific parameters of the CDP damage plasticity model, where ψ is the expansion angle, ϵ is the flow potential offset value, $f_{b0}/f_{c0}$ is the ratio of biaxial ultimate compressive strength to uniaxial ultimate compressive strength, $K_{c}$ is the invariant stress ratio, and μ is the viscosity coefficient.

For the concrete and steel tube, the reduced integral element (C3D8R) is adopted, which can avoid shear self-locking [25]. The truss element (T3D2) is for prestressed reinforcement. The contacts between the steel tube and concrete and between segments are all surface-to-surface contact. The relative slip between the steel tube and concrete and between segments is small. The “penalty friction” is for the Abaqus model, and the friction coefficients are 0.6 and 0.4 [26]. The radial restraint of the steel tube to concrete and the opening and closing of joints are defined as “hard contact”. Tie contacts are used between the steel pipe and the energy-dissipating element, as well as between the floor beam and the steel pipe.

Meshing is closely related to the type of element used. Mesh sensitivity analysis revealed that the linear red integral element used had only one integration point in the center of the component, so there was an “hourglass” problem. Therefore, finer meshes need to be divided to overcome the hourglass phenomenon, and at least four meshes need to be divided in the thickness direction, then divided by 1/10 of the width of the steel pipe. In the experimental calculation process, it is found that the smaller the grid, the longer the calculation time, the larger the grid, and the greater the error of the calculation result. Considering the calculation efficiency and accuracy, the overall grid density of the steel pipe, concrete, energy-dissipating elements, and steel strands is 200 mm, but in order to capture the local damage caused by swaying of the component, the grid size of some key parts (such as the connection between the energy-dissipating elements and the steel pipe, the bottom of the component, etc.) is 100 mm [27,28].

**Figure 7:**
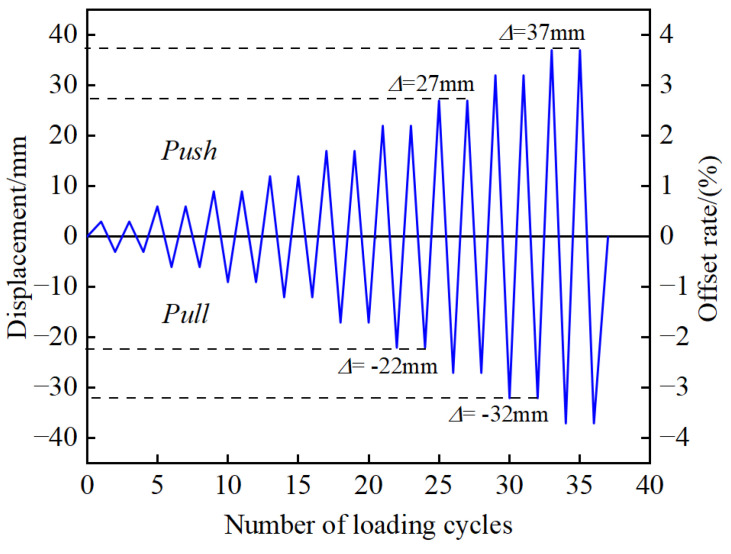
Displacement loading scheme.

The specimen is loaded by low-cycle reciprocating. The loading position is 100 mm from the top of the top segment. The bottom of the pier column is fully constrained to form a cantilever structure. The loading mode is displacement-controlled loading. The displacement amplitude of each stage is cycled forward and backward twice, that is, the loading displacement increases from 3 mm to 62 mm in turn. The loading scheme is shown in Figure 7.

**Figure 8:**
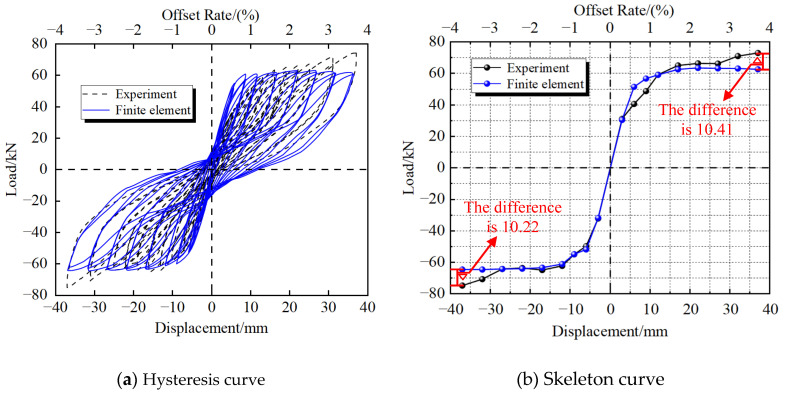
Diagram of the mechanical behavior of simulation results and test results.

Figure 8 shows the mechanical behavior of the PSCFSTBP using external energy-dissipating devices in simulation and testing. Among them, the skeleton curve of the finite-element numerical simulation result is taken as the average of two cycles per stage. It can be seen from the figure that when the displacement reaches the maximum value, the lateral maximum bearing capacity, equivalent stiffness, and energy dissipation capacity of the finite-element model are not significantly different from the test results, and the overall consistency of the curve is good, only when the pier top offset rate reaches the maximum; the peak bearing capacity difference is about 10kN, which is caused by the test steel constitutive not considering the strengthening stage, and the difference is within a reasonable range [29].

**Figure 9:**
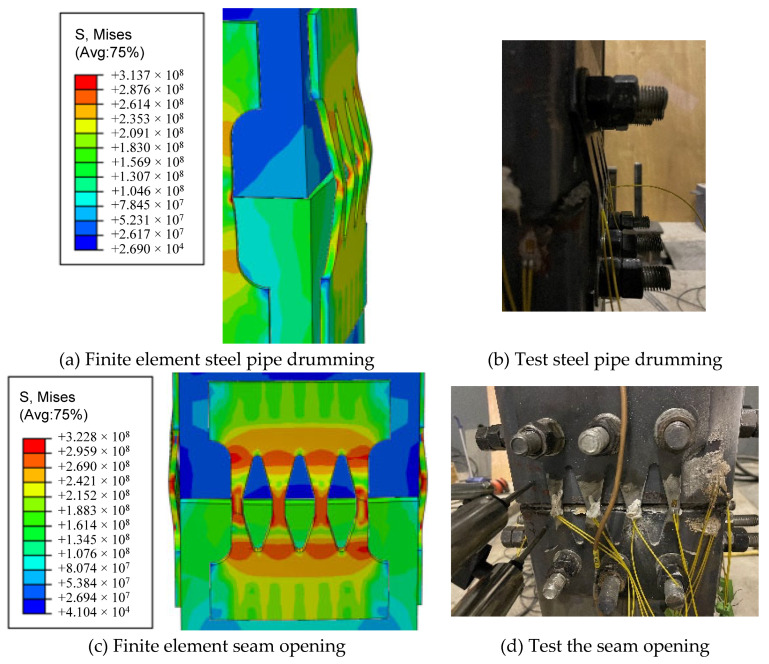
Comparison of stress distribution and deformation.

Table 3 shows a comparison of the three evaluation indicators. It can be seen that the different rates of lateral bearing capacity, initial stiffness, and energy dissipation capacity are 8.7%, 9.1%, and 5.9%, respectively, and the error is less than 10% [30,31]. Within a reasonable range, Figure 9 shows the deformation and stress distribution of the finite element, which is in good agreement with the experimental results. It can be seen that the finite-element model established in this paper has good reliability. It can be considered that the stress performance of the PSCFSTBP can be reasonably simulated, and subsequent simulation calculations can be carried out based on this.

As for the difference between the numerical simulation results and the experimental results, the main reason is due to the control of the lagging simulation. The loading part of the simulated hysteresis curve basically coincides with the loading part of the experiment. However, because the actual damage of concrete is affected by environmental and human factors, it will be different from the damage factor calculated in theory, resulting in the difference between the unloading part of the simulated hysteresis curve and the experiment. At the same time, because the constitutive model of reinforcement materials cannot simulate the negative stiffness effect after the ultimate strength, there is a certain difference between the calculated results and the test results.

### 3.2. Establishment of PSCFSTBP Finite-Element Model of External AEDD

Since the three-dimensional solid model in ABAQUS 6.14-4 can intuitively and accurately simulate the test process and present the results, this paper conducts numerical simulation on the PSCFSTBP without an energy dissipation device, the PSCFSTBP of external arch steel plate, the PSCFSTBP of the external vertical steel plate, and the PSCFSTBP of the external AEDD based on this analysis software.

**Figure 10:**
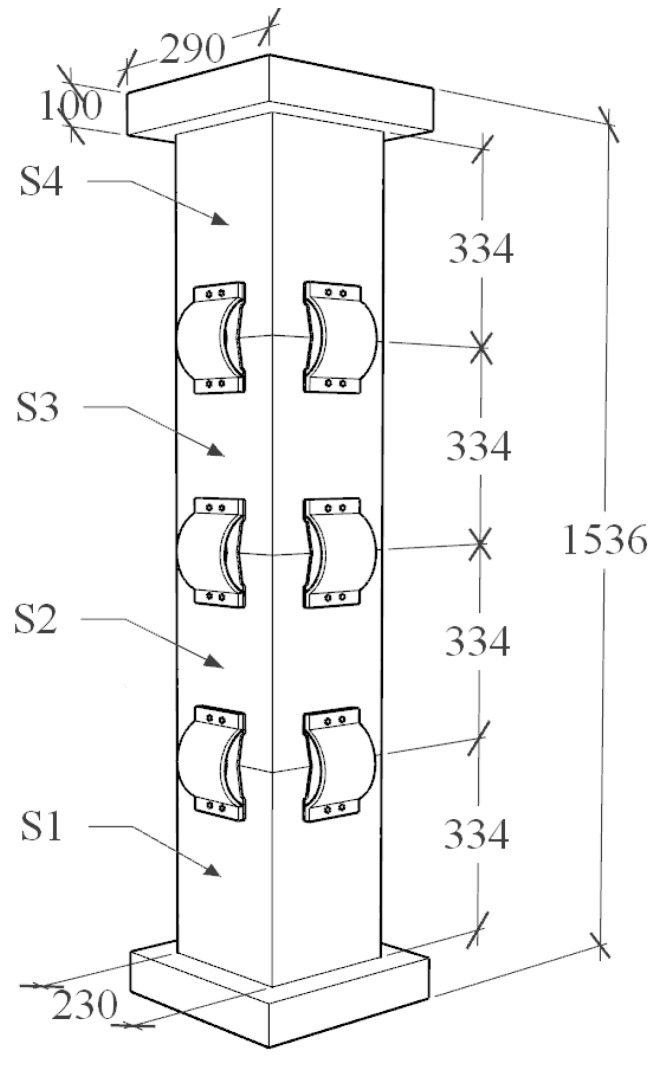
Structure of precast assembled pier test piece (unit: mm).

Using the ABAQUS 6.14-4 finite-element analysis platform, the PSCFSTBP of the above external different energy-consuming devices was established in turn, and the models were named no energy dissipation device (NEDD) specimens, external arched steel plate (EASP) specimens, external vertical steel plate (EVSP) specimens, and arch energy dissipation device (AEDD) specimens. The pier is composed of three parts: bearing foundation, pier body, and pier cap, of which the pier body has 4 prefabricated steel pipe restraint concrete segments of equal height, named S1, S2, S3, and S4 from bottom to top. Each test piece adopts a rectangular section. The section size is 230 mm × 230 mm, the height of each segment is 334 mm, and the effective pier height of the test piece is 1486 mm. The center of the pier column is reserved with a PVC prestressed rib pipe, the segment is connected by an unbonded post-tensioned steel strand located in the pipeline, the upper end is anchored to the center of the pier cap, the lower end is anchored to the bearing foundation, the initial tension force of the prestressed steel strand is 200kN, and the axial compression ratio of the specimen is 0.2. The restrained steel pipes of each section are made of Q345 steel and filled with C40 concrete. Other design parameters and simulation methods of the model are the same as in Section 3.1, and the structure and dimensions of the AEDD specimen are shown in Figure 10.

**Figure 11:**
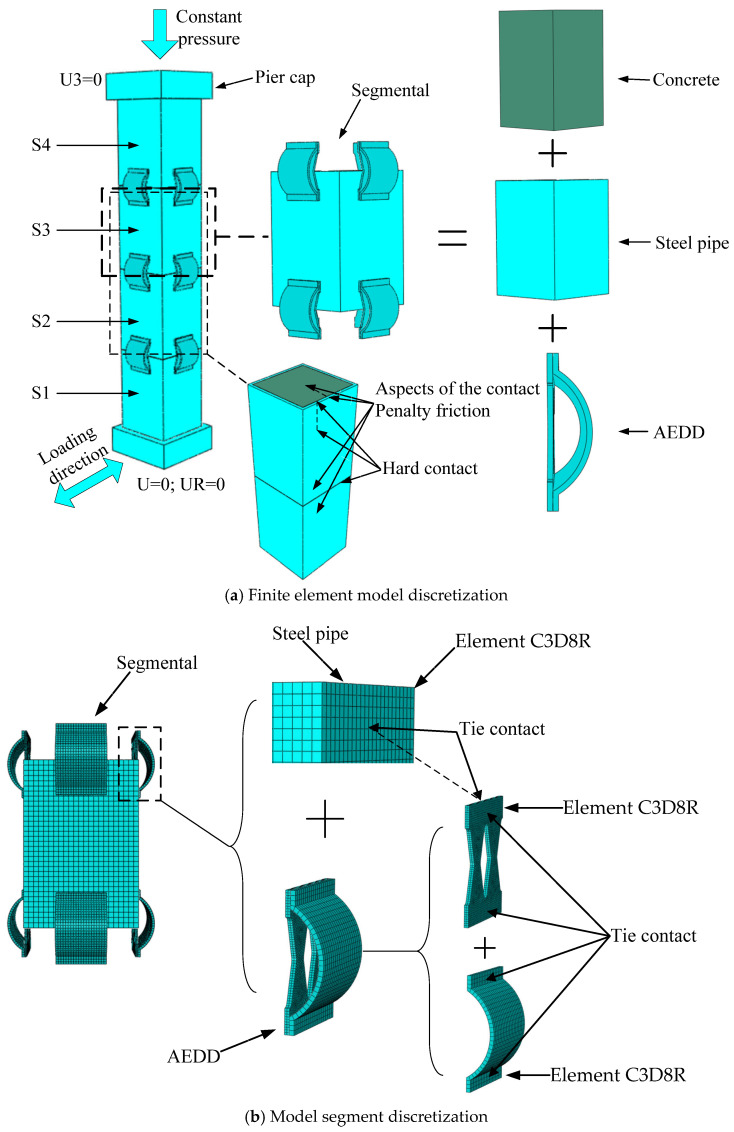
Finite-element model of PSCFSTBP with AEDD.

The vertical steel plate and arched steel plate of the AEDD are simulated by an eight-node linear hexahedral element C3D8R, the constitutive relation of which is based on the Chaboche nonlinear follow-up strengthening constitutive model [32]. Tie contact is used between the vertical steel plate and the arched steel plate of the AEDD and between the vertical steel plate and the steel pipe. In addition, as the prestressed reinforcement is unbonded, the prestressed reinforcement is divided into three parts. The part extending into the foundation and pier cap is set as a nested connection, and the middle part is not treated to simulate the unbonded state of the prestressed reinforcement. The analysis step of the model includes three steps. The first step is to apply the prestress with the cooling method [33]. The second step is to reload the axial pressure, the axial force is to simulate the force of the bridge superstructure on the pier. The third step is to apply low-cycle reciprocating loading in the horizontal direction. Finally, all degrees of freedom of the pier bottom will be constrained, and no degrees of freedom of the pier top will be constrained, so that the pier bottom is fixed and the pier top is free, forming a cantilever structure. In order to obtain the reaction force at the bottom of the pier, a reference point is established at the center of the foundation base. The coupling method is used to couple the center of the foundation base and the reference point, and constrain the translation and rotation degrees of freedom of the reference point in three directions to achieve the consolidation of the pier bottom. The PSCFSTBP model of the external AEDD is shown in Figure 11.

**Figure 12:**
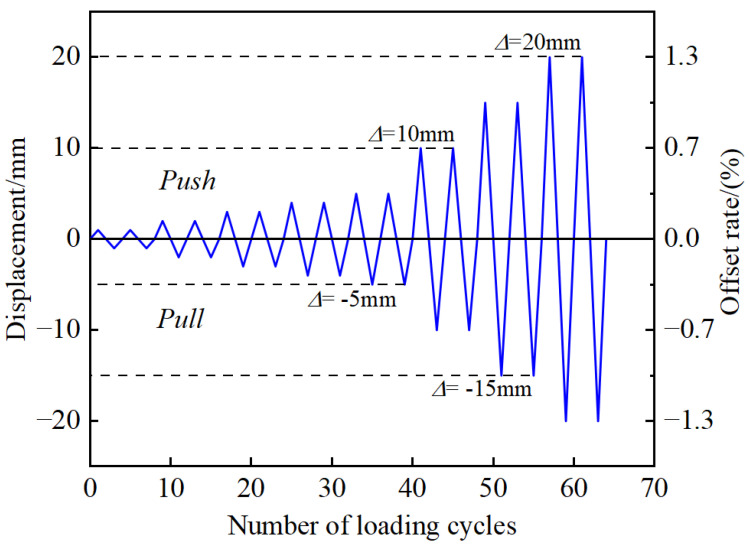
Loading regime.

The loading mode of each specimen is low-cycle reciprocating loading, the loading position is at the side center of the pier cap, and the bottom of the pier column is fully constrained to form a cantilever structure. The loading mode adopts displacement control, and the horizontal offset rates of the pier top are 0.07%, 0.13%, 0.20%, 0.27%, 0.34%, 0.67%, 1.01%, and 1.35%, respectively. Each stage of loading cycles twice, and the loading curve is shown in Figure 12.

## 4. Structural Optimization Analysis of External Arch Device

In order to explore the “optimal” energy dissipation device external to the PSCFSTBP, this section will use the numerical analysis results of the finite-element model and make a comparative analysis from the classical perspectives of the hysteresis curve, skeleton curve, cumulative energy dissipation curve, residual displacement curve, and stiffness degradation curve.

### 4.1. Hysteresis Curve

**Figure 13:**
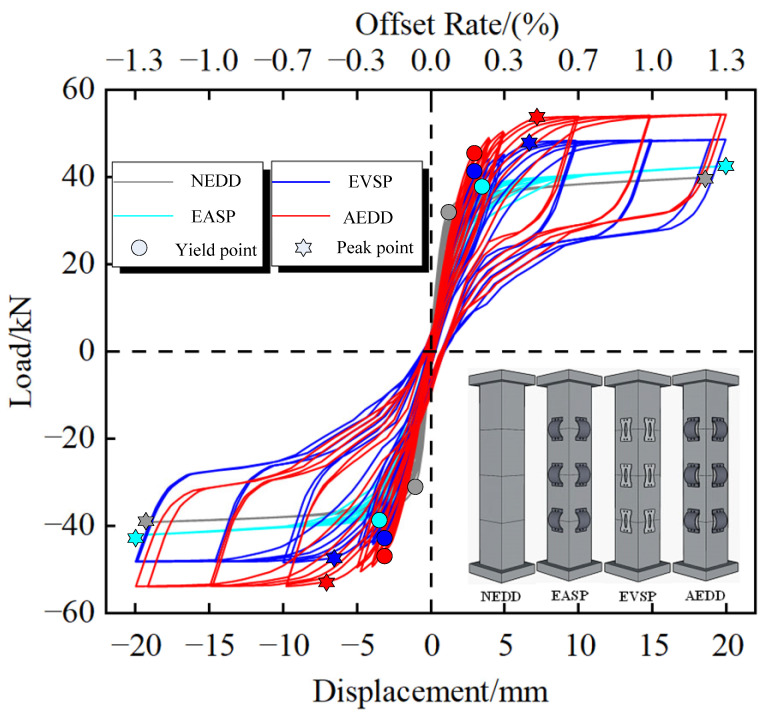
Hysteretic curves of different piers.

Figure 13 shows the hysteresis curves of the PSCFSTBP with different external energy-consuming devices. The hysteresis curve refers to the relationship curve between the load and displacement obtained under the action of horizontal cyclic load. It can be seen from the figure that at the initial stage of displacement loading, the test pieces all develop linearly, indicating that they are in the elastic stage at this time. The initial stiffness of the AEDD test pieces is greater than that of the NEDD test pieces, EASP test pieces, and EVSP test pieces. This is due to the external AEDD of the AEDD test pieces. Compared with the external arch steel plate or vertical steel plate, the combined effect of the two is better, so the integrity of the AEDD test pieces is better and the initial stiffness is greater.

It can be seen that all the test pieces are symmetrical in both positive and negative directions. The hysteresis area of the NEDD test piece and the EASP test piece is small, so the energy consumption capacity is poor. The reason for the poor energy consumption of the EASP test piece is that the test piece only has an arch steel plate instead of a vertical steel plate. When the arch steel plate is placed on the PSCFSTBP, the opening and closing of the joint under the action of horizontal force will cause insufficient longitudinal energy consumption, which is easy to cause damage to the arch steel plate. It shows that the vertical steel plate has a greater effect on energy consumption. The hysteresis curves of the AEDD and EVSP specimens are similar to flag-shaped curves. The curves are relatively full, and the hysteresis loop area is large, so the energy dissipation capacity is strong, which can absorb more energy from earthquakes. The reason why the AEDD test piece consumes more energy than the EVSP test piece is that the arch device can consume energy at multiple levels. The vertical steel plate of the AEDD test piece is the main energy-consuming steel plate, but the arch steel plate also improves the energy consumption to a certain extent, which also proves that the energy consumption of the EASP test piece is greater than that of the NEDD test piece.

It can also be seen that the residual displacement of each specimen remains at a small level (NEDD test piece, EASP test piece, EVSP test piece). The maximum horizontal bearing capacities of the AEDD specimens are 39.05kN, 42.57kN, 48.65kN, and 54.44kN, respectively, which shows that for improving the lateral bearing capacity of members, setting the AEDD at the PSCFSTBP joints is better than external arch steel plates or vertical steel plates. For the hysteresis curve of the AEDD specimen, there is an obvious phenomenon of “pinching”, which indicates that the AEDD plays a certain role in the reset of piers, and the “pinching” effect is caused by the slippage between pier segments and the shear deformation of the AEDD energy dissipation steel plate.

### 4.2. Skeleton Curve

**Figure 14:**
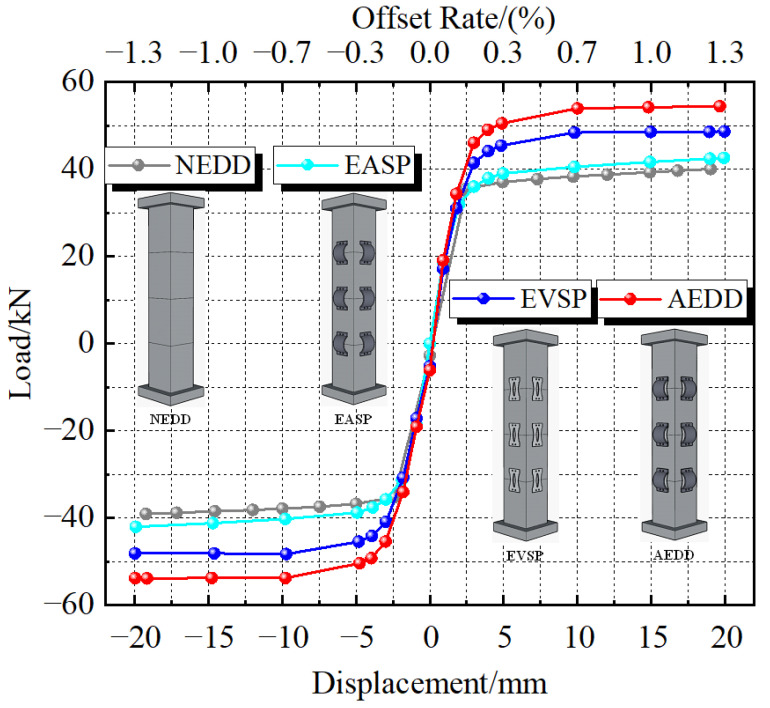
Skeleton curves of different types of piers.

Figure 14 shows the skeleton curves of the PSCFSTBP with different external energy-consuming devices, and Table 4 shows the characteristic values of the skeleton curves. The skeleton curve is formed by the peak point connection of each level of load in the hysteresis curve, which can directly reflect the changes in load and displacement. It can be seen from the figure that the bearing capacity of each specimen increases with the increase in displacement loading in both the positive and negative loading directions. At the initial stage of loading, each specimen is in the elastic stage, and the curve is linear. However, because the slope of the AEDD specimen is the largest, its initial stiffness is the largest, and its integrity is the best. Therefore, when the value of the same loading displacement is equal, the bearing capacity of the NEDD specimen, EASP specimen, and EVSP specimen is smaller than that of the AEDD specimen. With the loading of displacement, the stiffness of each specimen decreases, indicating that the pier has entered the elastoplastic stage, but the lateral bearing capacity of the AEDD specimen is still greater than that of other types of specimens. The maximum horizontal bearing capacities of the NEDD, EASP, EVSP, and AEDD specimens are 39.05kN, 42.57kN, 48.65kN, and 54.44kN, respectively, and the bearing capacities of the external arch steel plate, external vertical steel plate, and external AEDD are increased by 9.01%, 24.58%, and 39.41%, respectively. It shows that the lateral bearing capacity of the external AEDD at the PSCFSTBP joint is optimal.

### 4.3. Cumulative Energy Consumption

**Figure 15:**
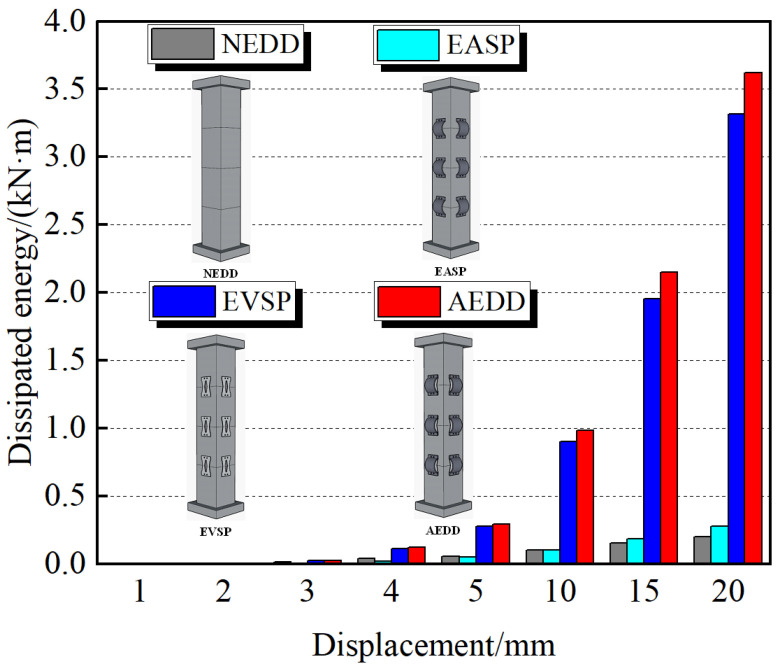
Columnar diagram of the cumulative energy consumption of different forms of piers.

Figure 15 shows the cumulative energy consumption histogram of the PSCFSTBP with different external energy-consuming devices. Cumulative energy consumption refers to the sum of the areas enveloped by the hysteresis curve at each loading level. Generally, the larger the value, the stronger the energy consumption capacity. It can be seen from the figure that the cumulative energy consumption capacity of the AEDD test piece is greater than that of the NEDD test piece, EASP test piece, and EVSP test piece. When each test piece reaches the maximum displacement, the cumulative energy consumption of the NEDD test piece, EASP test piece, EVSP test piece, and AEDD test piece is 200kN · mm, 280kN · mm, 3316kN · mm, and 3622kN · mm, respectively. The cumulative energy consumption of the EASP test piece, EVSP test piece, and AEDD test piece is about 1.4 times, 16.6 times, and 18.1 times the cumulative energy consumption of the NEDD test piece. It can also be seen that, with the increasing displacement loading, the increase in the AEDD specimen is the largest among the specimens, which indicates that the external AEDD is superior to the external arch steel plate and the vertical steel plate in improving the cumulative energy consumption capacity of the PSCFSTBP.

### 4.4. Residual Displacement

**Figure 16:**
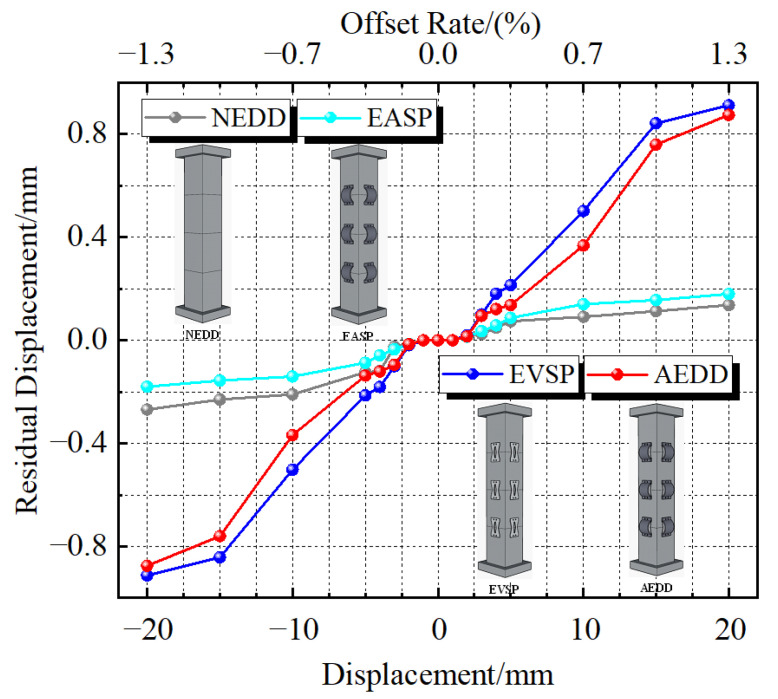
Residual displacement change curves of different forms of piers.

Figure 16 shows the PSCFSTBP residual displacement change curve of the different external energy-consuming devices. Residual displacement refers to the deformation that cannot be recovered after reciprocating displacement loading, also known as permanent deformation, which is an important reference index for the recoverability of the pier shaft. It can be seen from the figure that the residual displacement of the AEDD specimen and the EVSP specimen is relatively large, while that of the NEDD specimen and EASP specimen is relatively small. The reason is that the AEDD specimen and EVSP specimen are equipped with external energy-consuming devices, and it is relatively difficult to recover due to the “drag” of the energy-consuming devices after displacement loading. As for the EASP specimen, which is only equipped with external arch steel plates, the arch steel plates will be damaged and cannot work due to insufficient longitudinal energy consumption. The specimen is similar to PSCFSTBP without an energy dissipation device, so its residual displacement is small. The residual displacement of the AEDD specimen is smaller than that of EVSP specimen, which indicates that the arch steel plate has a certain self-resetting effect. Although the residual displacement of the NEDD specimen and EASP specimen is small, they are “sacrificing” the energy consumption capacity as a cost, and the residual displacement of each specimen is not more than 1 mm, and the offset rate is not more than 1%, which is easy to repair after the earthquake. Relatively speaking, the PSCFSTBP effect of external AEDD is the most practical.

### 4.5. Stiffness Degradation

**Figure 17:**
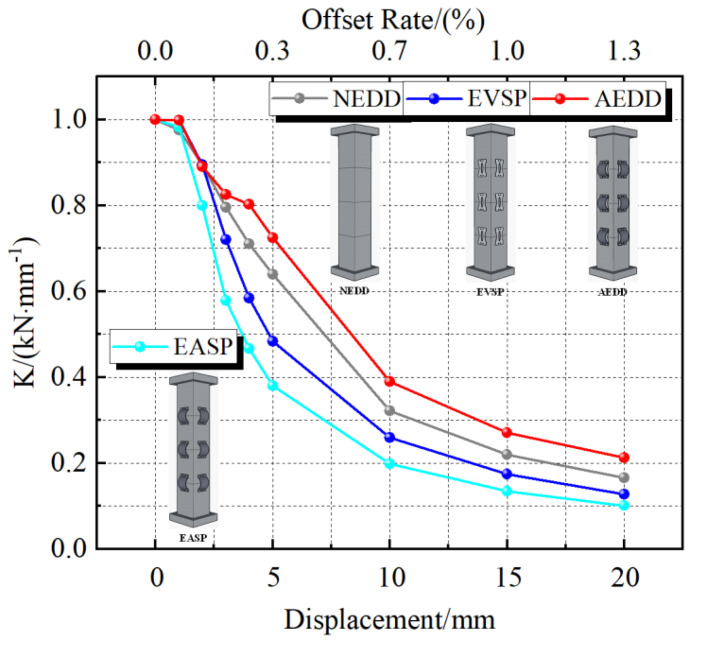
Stiffness degradation curves of different forms of piers.

Figure 17 shows the PSCFSTBP stiffness degradation curve of the different external energy-consuming devices. Stiffness degradation refers to the phenomenon that the displacement of the peak point increases with the increase in the number of cycles when the same peak load is maintained under the action of cyclic repeated load. The magnitude can be expressed as the ratio of the equivalent stiffness to the initial stiffness. It can be seen that the stiffness degradation of each curve is continuous and stable, and there is no sudden stiffness change damage. In the initial displacement loading stage, the stiffness degradation rate of each specimen is fast, and with the displacement loading, the degradation rate of each specimen decreases. It can also be seen from the figure that the degradation rate of the EASP specimen is the largest, because the initial stiffness of the EASP specimen is large, and the arch steel plate is damaged when the specimen is loaded, resulting in rapid degradation. When loading to the maximum displacement, the equivalent stiffness of the NEDD, EASP, EVSP, and AEDD specimens decreases to 16.6%, 10.1%, 12.6%, and 21.3% of the initial stiffness, respectively. It can be found that the AEDD specimens have the lowest stiffness degradation, which indicates that the AEDD can slow down the stiffness degradation. The reason for stiffness degradation should be that the joint of the segment keeps opening and closing, and plastic deformation reduces the stiffness of the specimen during the loading process.

It can be seen that the PSCFSTBP of the external AEDD has the largest initial stiffness, the strongest energy dissipation capacity, the largest lateral bearing capacity, and the slowest stiffness degradation compared with the PSCFSTBP of the external arch steel plate and the external vertical steel plate. Although the residual displacement is not the minimum, it is still within a reasonable range. All in all, the AEDD is optimal.

## 5. Multi-Level Energy Consumption and Local Replacement of AEDD

Compared with other energy-consuming devices, the AEDD also has two features, namely, multiple energy consumption and local replacement after an earthquake.

### 5.1. Multi-Level Energy Consumption

**Figure 18:**
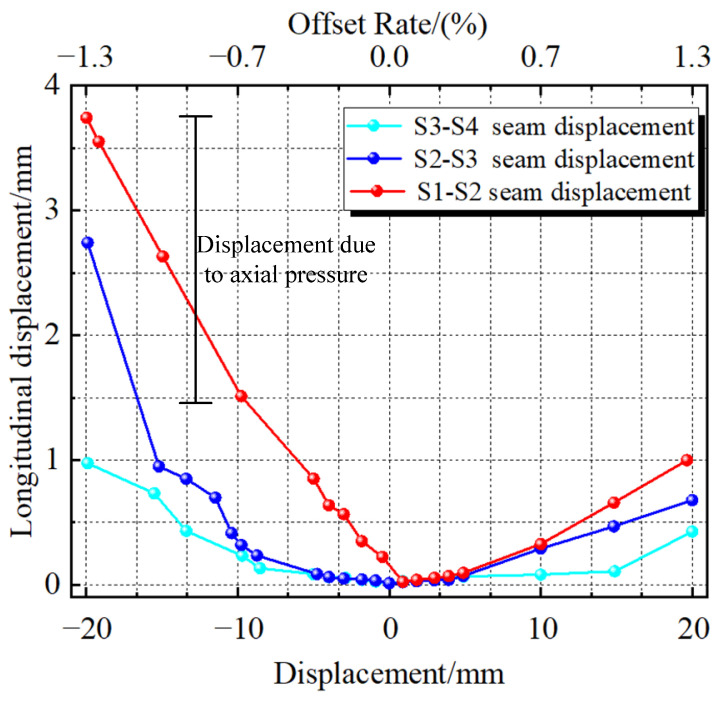
Longitudinal displacement of arched steel plates of each joint arch energy-consuming device.

Figure 18 shows the longitudinal displacement change in the arch steel plate with the external AEDD at each joint of the PSCFSTBP tensile side. The longitudinal displacement change in the arch steel plate is caused by the opening and closing of each segment. It can be seen from the figure that each joint has a longitudinal displacement, indicating that each joint can consume energy. The longitudinal displacement of the S1-S2 joint is the largest, that of the S2-S3 joint is the second, and that of the S3-S4 joint is the smallest, which indicates that the longitudinal displacement at the joint of the pier increases gradually from top to bottom, and also indicates that the energy of the AEDD dissipation earthquake at the joint increases gradually from top to bottom. Therefore, it can be seen that the AEDD at the S1-S2 joint contributes more to energy consumption.

The results also show that the longitudinal displacements of the arch steel plate have changed. It can be seen that there are displacements in the horizontal direction, indicating that it has a certain energy consumption capacity in the horizontal and vertical directions. As the vertical steel plate is a mild steel with a low yield point, it is used as the first-level energy-consuming component. The arch steel plate has a high yield point and can be used as the second-level energy-consuming component. The yield process of the Section 3.1 and Chapter 1 AEDD can be reflected here. As for the asymmetry of the two sides of the longitudinal displacement, the reason is the axial pressure.

### 5.2. Partially Replaceable

**Figure 19:**
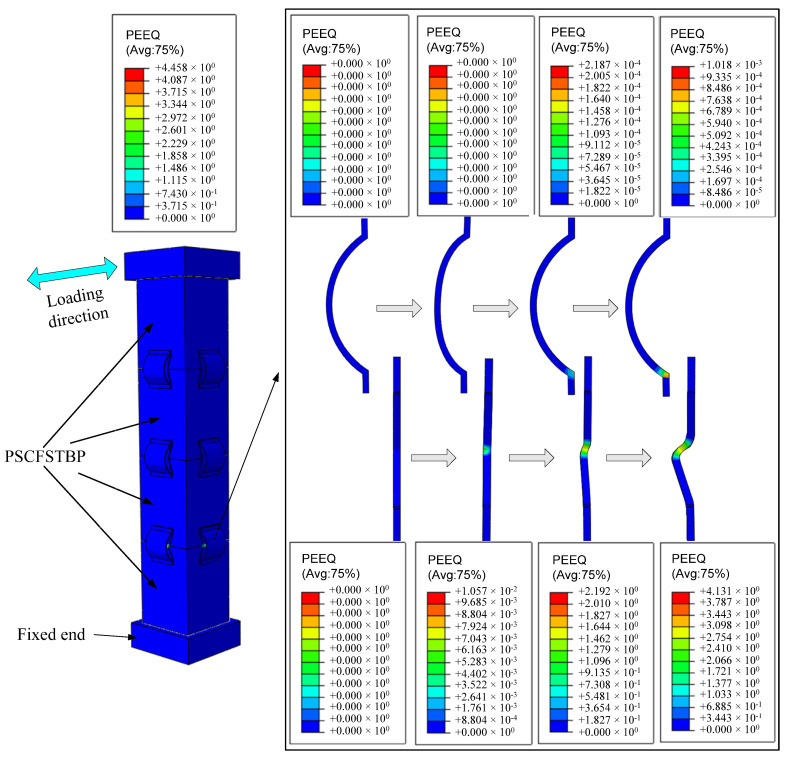
PEEQ (equivalent plastic strain nephogram).

Figure 19 shows the PSCFSTBP equivalent plastic strain nephogram [34,35] of the external AEDD. It can be seen from the figure that the steel tube of the pier has not yielded, and there is no obvious buckling. It can be seen from the AEDD nephogram of the bottom joint at the tension side that, at the initial stage of loading, the arch steel plate and vertical steel plate of the AEDD did not yield. When the horizontal deviation rate of the pier top reaches 7%, the bottom joint at the tension side of the pier opens, and the longitudinal tension causes the arch steel plate and vertical steel plate to be stretched. At the same time, the opening in the middle of the low-yield vertical steel plate yields. When the pier top deviation rate reaches 1%, plastic strain occurs at the arch angle under the arch steel plate. At the same time, the plastic strain and strain area of the weak area in the middle of the vertical steel plate increase. When the pier top offset reaches 1.3%, the vertical steel plate deformation is larger, and the plastic strain of the arch angle of the arch steel plate increases. It can be seen that the damage to the PSCFSTBP is mainly concentrated on the AEDD, and the damage is controllable. Under the action of horizontal force, the vertical steel plate of the AEDD preferentially produces plastic strain, participates in energy consumption, and the arch steel plate finally yields, so it can be used as the energy consumption of the second level and the stiffness reserve at the joints. Therefore, in the low-intensity area, the PSCFSTBP of the external AEDD can only be replaced because the arch steel plate may not reach the yield strength. The arch steel plate that can still be used does not need to be replaced as a whole, which improves the repair speed after the earthquake, reduces the repair difficulty, and is more economical and reliable.

**Figure 20:**
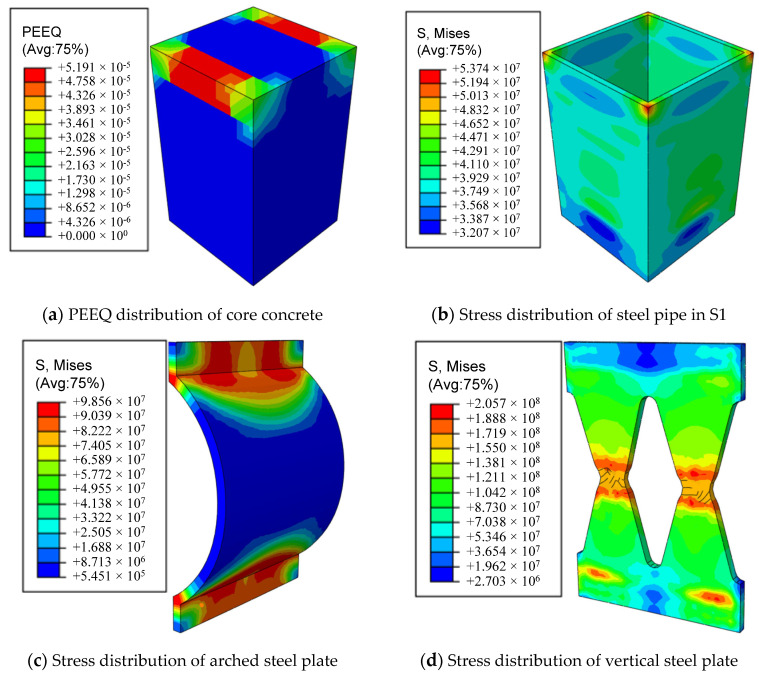
Stress distribution of PSCFSTBP with external AEDD.

Figure 20 shows the stress cloud and equivalent plastic strain cloud corresponding to the AEDD set on the tensile side of the S1 section steel pipe, concrete, and S1-S2 joint of the model when the pier roof offset rate is the largest, so as to judge the degree of controllable damage and the feasibility of rapid repair of this type of pier after an earthquake. It can be seen from the figure that the steel pipe of the S1 section does not reach the yield stress, no obvious drum deformation occurs, and the plastic damage range of concrete is small. The vertical steel plates of the AEDD on the tensile side of the S1-S2 joint have yielded, and the vertical steel plates have irreparable plastic deformation caused by tension, while the arched steel plates have no obvious plastic strain, and only partially yield at the arch corners, and the yield is caused by the participation of the arched steel plate in the longitudinal energy consumption. It can be seen from the above that the PSCFSTBP deformation damage and energy dissipation are mainly concentrated on the AEDD at the joint, so the pier body will not be seriously damaged. In addition, since the AEDD is placed externally at the seam of the PSCFSTBP, it ensures that the AEDD can be easily replaced after the earthquake. This echoes above.

## 6. Conclusions

(1) Under horizontal seismic force, the vertical steel plate of the AEDD will deform before the vertical steel plate reaches the yield point, and the damage is concentrated in the weak area of the middle opening of the vertical steel plate. The damage is controllable, and the arch steel plate has a small deformation. With the continuous increase in the horizontal force, the arch corner and the middle point of the arch steel plate enter the yield state.

(2) The PSCFSTBP of the external AEDD can improve the initial stiffness (2.5%~10.4%), lateral bearing capacity (11.9%~39.4%), and energy dissipation capacity (1.1~18.1 times) compared with the PSCFSTBP of the other three construction forms. In addition, when the displacement is loaded to the maximum, the PSCFSTBP equivalent stiffness of the external AEDD degrades to 21.3% of the initial stiffness, the residual displacement is always kept within 1 mm, and the offset rate is not more than 1%, which can achieve rapid repair after the earthquake. Based on comprehensive analysis, it is recommended to set the external AEDD to PSCFSTBP.

(3) The AEDD of each joint of the PSCFSTBP can dissipate energy, and the lower the joint position of the segment is, the more energy the AEDD dissipates. The arch steel plate of the AEDD has the capacity for energy dissipation, which can be used as the energy dissipation of the second level and the stiffness reserve at the joints.

(4) When the offset rate is low, the components of the AEDD at each joint of the PSCFSTBP will present multi-stage yields. Therefore, it can be predicted that under the seismic action of different strengths, the damaged components of the energy-consuming components can be replaced in a targeted way, so as to achieve more flexible and economic replacement of the energy-consuming components.

(5) The PSCFSTBP of the external AEDD in this paper still has the following limitations: (1) There is no experimental study, only the feasibility of this device is explored and verified, and it must be further explained by experiments; (2) Although the relevant data are analyzed through finite-element numerical simulation, there is a lack of research on the influence of design parameters, and subsequent parameter modeling and experimental exploration need to be changed; (3) Only four segments of the PSCFSTBP were numerically simulated, and various other segments must be explored for analysis.

## Figures and Tables

**Table 1:** Material property test results.

Material	Model	The Yield Strength *f*_y_/MPa	Ultimate Strength *f*_u_/MPa	Compressive Strength *f*_c_/MPa	Modulus of Elasticity E_s_/(×10^4^ MPa)
Steel	Q235	276.6	474.1	-	20.4
	Q345	381.7	580.2	-	20.8
Concrete	C40	-	-	42.6	3.26

**Table 2:** Parameters of concrete plastic damage model.

ψ	ϵ	$f_{b0}/f_{c0}$	$K_{c}$	μ
30	0.1	1.16	0.6667	0.0005

**Table 3:** Comparison between simulation results and test results.

Compare the Item	Lateral Bearing Capacity/(kN)	Equivalent Stiffness/(kN·mm^−1^)	Energy Dissipation/(kN·m)
The test results	74.1	1.1	15.3
The simulation results	67.6	1.0	16.2
Difference rate	8.7%	9.1%	5.9%

**Table 4:** Performance turning point of skeleton curve.

Specimen	The Yield Strength/kN	The Yield Displacement/mm	Peak Bearing Capacity/kN	Peak Displacement/mm	Ductility Coefficient
NEDD	38.32	9.72	40.02	19.03	1.96
EASP	40.50	9.80	42.57	19.93	2.03
EVSP	48.39	9.80	48.65	19.95	2.04
AEDD	53.92	10.00	54.44	19.65	1.97

## Data Availability

All data, models, or code supporting the results of this study are available from the corresponding authors upon reasonable request.
